# ACK-MSCKF: Tightly-Coupled Ackermann Multi-State Constraint Kalman Filter for Autonomous Vehicle Localization

**DOI:** 10.3390/s19214816

**Published:** 2019-11-05

**Authors:** Fangwu Ma, Jinzhu Shi, Yu Yang, Jinhang Li, Kai Dai

**Affiliations:** State Key Laboratory of Automotive Simulation and Control, Jilin University, Changchun 130022, China

**Keywords:** autonomous vehicles, visual–inertial odometry, Ackermann error state measurements, sensor fusion

## Abstract

Visual-Inertial Odometry (VIO) is subjected to additional unobservable directions under the special motions of ground vehicles, resulting in larger pose estimation errors. To address this problem, a tightly-coupled Ackermann visual-inertial odometry (ACK-MSCKF) is proposed to fuse Ackermann error state measurements and the Stereo Multi-State Constraint Kalman Filter (S-MSCKF) with a tightly-coupled filter-based mechanism. In contrast with S-MSCKF, in which the inertial measurement unit (IMU) propagates the vehicle motion and then the propagation is corrected by stereo visual measurements, we successively update the propagation with Ackermann error state measurements and visual measurements after the process model and state augmentation. This way, additional constraints from the Ackermann measurements are exploited to improve the pose estimation accuracy. Both qualitative and quantitative experimental results evaluated under real-world datasets from an Ackermann steering vehicle lead to the following demonstration: ACK-MSCKF can significantly improve the pose estimation accuracy of S-MSCKF under the special motions of autonomous vehicles, and keep accurate and robust pose estimation available under different vehicle driving cycles and environmental conditions. This paper accompanies the source code for the robotics community.

## 1. Introduction

Providing a real-time accurate vehicle position is one of the key technologies to enhance the active safety of advanced driver assistance systems (ADASs) and achieve autonomous driving [1]. Wheel odometry is a fundamental method in most autonomous vehicles, owing to low cost and high reliability. It uses the information from the proprioceptive sensors, including the drive motor encoder sensor and steering wheel angle sensor, to estimate the position relative to a starting location with the method of dead-reckoning, based on the kinematics model. It exhibits the characteristic of high positioning accuracy in short-term operation. However, it is sensitive to systematic and non-systematic errors [2]. The accumulated errors will result in a drift in position estimation gradually over time. Therefore, it is necessary to fuse data from other sensors to improve positioning accuracy [3]. The fusion of the Global Navigation Satellite System (GNSS) and the Inertial Navigation System (INS) can provide high-accuracy global positioning [4]. However, the performance degrades quickly in GPS-denied environments. The positioning technology based on the Light Detection and Ranging (LIDAR) odometry [5,6] has the advantages of algorithm maturity, high precision and real-time performance, but its application in vehicles is limited due to the high cost of LIDAR. 

On the other hand, visual odometry (VO), which adopts visual sensors, is gaining significant interest by the robotics community owing to the abundant perceptive information, small size and low cost [7,8,9]. A detailed review of the VO methods can be found in the literature [10,11,12]. While VO algorithms undergo a lack of robustness when confronted with motion blur, low texture scenes, illumination changes, sharp turns, partial or full occlusions and the influence of dynamic objects [13], visual-inertial odometry (VIO) [14,15,16,17] algorithms can significantly improve the performance by fusing low-cost IMUs, which attract much attention in the field of micro air vehicles (MAVs). For vehicle positioning, Ramezani and Khoshelham [18] propose a stereo Multi-State Constraint Kalman Filter (Stereo-MSCKF) using MSCKF [19], which shows a promising potential to provide positioning with sub-meter accuracy in short periods of GNSS signal loss. By far, VIO algorithms mainly consist of EKF-based methods, including MSF [20], ROVIO [21], MSCKF [19], Stereo-MSCKF [18], S-MSCKF [22], R-VIO [23] and optimization-based methods including the Open Keyframe-based Visual-Inertial SLAM (OKVIS) [24], VINS-Mono [25], PL-VIO [26] and Basalt [27]. Delmerico and Scaramuzza [28] performed a thorough evaluation of open source VIO, suggesting that EKF-based algorithms display better efficient performance, whereas optimization-based algorithms provide higher accuracy. Therefore, the trade-off between efficiency and accuracy in practical applications should be considered in order to choose an appropriate off-the-shelf VIO algorithm.

However, VIO algorithms provide enormous challenges for autonomous vehicle localization. Firstly, the outdoor traffic scene is completely uncontrolled. The scene can be cluttered, and thus contains a number of moving objects, such as pedestrians and vehicles. Therefore, VIO must be able to work precisely and robustly in such challenging situations to ensure the safety of pedestrian and passenger. Moreover, the on-board computer is an embedded system with limited computational resources. Hence, VIO should possess low computational complexity for the convenience of in-vehicle deployment [29]. Finally and most importantly, autonomous vehicles usually move on an approximately planar environment, and the motion is constrained to be of either constant local acceleration or no rotation. Under these special motions, VIO is subjected to additional unobservable directions [30], which indeed leads to larger positioning errors.

To address the above problems, there are some studies to integrate VIO algorithms with additional sources of information. VINS-RGBD [31] is proposed to fuse RGB-D and IMU data for small ground rescue robots, which overcomes the problem of scale unobservability mentioned in [30]. However, the use of the RGB-D sensor does not apply to the outdoor traffic scene in direct sunlight [32] for autonomous vehicles. He et al. [26] propose a tightly-coupled monocular VIO system, PL-VIO, by integrating more structured line features. The PL-VIO improves positioning accuracy, especially in uncontrolled outdoor scenarios. Nevertheless, the line detection and matching were the bottlenecks in the efficiency of the system. Zheng and Liu [33] propose an SE(2)-constrained pose parameterization optimization-based VIO for ground vehicles, which could obtain better accuracy under sharp-turn motion. Wu et al. [34] prove that the VIO scale, or two additional degrees of freedom (DOF) of global orientation, could become unobservable when ground mobile robots move with constant acceleration, or no rotation, respectively. They propose to fuse wheel odometry measurements into VIO, which significantly improves positioning accuracy under special motions. Dang et al. [35] propose a tightly-coupled VIO to fuse wheel encoder measurements by considering wheel slippage, which achieves great improvements in positioning accuracy. Huai and Huang [23] propose a robocentric VIO (R-VIO) by reformulating the system with respect to the IMU frame, and show that this system can resolve the scale unobservability issue under special motions without using additional sensors.

Although the above tightly-coupled VIO systems achieve high accuracy and robustness, they are sensitive to initialization [36] and require a higher computational burden due to the iterative optimal backend. Moreover, these methods are based upon differential drive robots or hand-held equipment. In contrast to the wide utilization of inertial measurements, KO-Fusion [37] is proposed to integrate Mecanum wheel motion constraint and the RGB-D sensor for small ground rescue robots, which improves the robustness under the fast and loopy motion. 

However, the use of the RGB-D sensor and Mecanum wheels does not directly apply to autonomous vehicles which usually run on the asphalt road in the outdoor high-light traffic scene. Moreover, its efficient performance should be further optimized for the convenience of in-vehicle deployment. As the on-road and off-road autonomous vehicles in the outdoor environment are mostly Ackermann steering robots [38], it is necessary to demonstrate in further research works whether the above methods are applicable to Ackermann steering robots.

Therefore, the purpose of this paper is to effectively utilize the wheel odometry and steering model of Ackermann steering robots to improve the pose estimation accuracy under special motions of autonomous vehicles, and to keep pose estimation available with accuracy and robustness under different vehicle driving cycles and environmental conditions. In view of the efficient performance, an open source EKF-based VIO is selected as the base of our work, i.e., S-MSCKF [22], which follows the same fundamental principle as Stereo-MSCKF [18]. We propose a tightly-coupled Ackermann Multi-State Constraint Kalman Filter, termed ACK-MSCKF, to fuse Ackermann error state measurements and S-MSCKF with tightly-coupled EKF-based mechanism. The main contributions of this paper are highlighted as follows:

(1) Conducting the formulation and implementation of a tightly-coupled visual-inertial odometry system, ACK-MSCKF, which significantly improves the pose estimation accuracy under the special motions of autonomous vehicles, and can allow the position and orientation estimation to remain accurate and robust under different vehicle driving cycles and environmental conditions.

(2) The rotation extrinsic parameters calibration method between vehicle coordinates and VIO coordinates is presented with a performance comparison.

(3) Facilitating the reproducibility of related research with the source code [39] of ACK-MSCKF publicly available.

(4) The performance of ACK-MSCKF is compared with the state-of-the-art open source stereo VIO, S-MSCKF and OKVIS [24] on the real-world datasets acquired by our experimental vehicle with both qualitative and quantitative analysis results.

The remainder of this paper is structured as follows: The formulation and implementation of the proposed approach are both introduced in Section 2, and then Section 3 exhibits corresponding experiments and results. Besides, the experimental results are demonstrated in Section 4. Finally, our conclusions are drawn in Section 5.

## 2. The Proposed Approach

**Figure 1:**
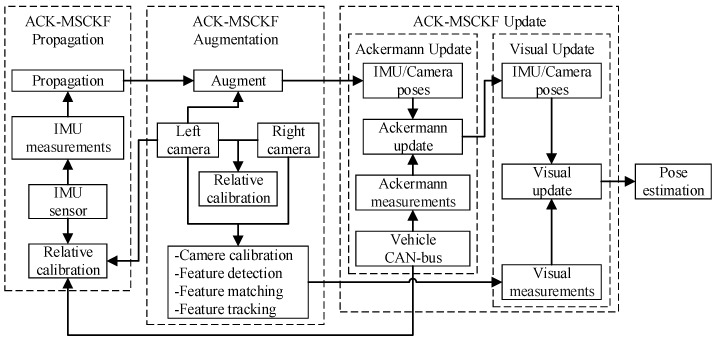
The basic workflow of Ackermann visual-inertial odometry (ACK-MSCKF).

The basic principle of ACK-MSCKF is shown in Figure 1. The process model in which the state, including parameters to be estimated, is propagated using IMU measurements. Then, the state is augmented with the new camera state of the left camera. Finally, the state is successively updated by Ackermann measurements and visual measurements.

### 2.1. Coordinate Systems and Notations

(1) Vehicle Coordinate System {B}. The {B} is fixed with the vehicle body. The origin of {B} lies on the automotive rear axle center. The *x*-axis and *y*-axis points to forward and left, respectively, following the right-hand rule.

(2) World Coordinate System {W}. The {W} has the same origin and axes as the {B} at the initial time.

(3) VIO Coordinate System {I}. The origin of {I} lies on the center of the stereo camera, which is installed in the front of the vehicle. The *x*-axis and *y*-axis points to upward and right, respectively, following the right-hand rule.

(4) Camera Coordinate System {C}. The origin of {C} lies on the center of the left camera. The *x*-axis and *y*-axis points to right and downward, respectively, following the right-hand rule.

(5) Inertial Coordinate System {G}. The origin of {G} is the same as that of {I} at the time of VIO initialized. The axes of {G} are obtained by calculation at the time of VIO initialized, and its *z*-axis is aligned with earth gravity.

In this paper, we adopt the JPL [40,41] quaternion conventions which are used in S-MSCKF to derive ACK-MSCKF and the Hamilton [42] quaternion conventions to implement rotation extrinsic parameters calibration.

### 2.2. Process Model and State Augmentation

The IMU state at the sampling time of IMU $t_{k}$ is defined as,

(1) XIk=[qGIkTbg,kTvGIkTba,kTpGIkTqICTpICT]T

where qGIk denotes the rotation quaternion from {G} to {I},vIkG and pIkG are the velocity and position of {I} in {G}, qIC and pIC both denote the exterior parameters between {I} and {C}. The symbols $b_{g,k}^{}$ and $b_{a,k}^{}$ are the IMU’s gyroscope and accelerometer biases, respectively.

The camera state at the sampling time of camera $t_{j}$ is defined as,

(2) XCj=[qGCjTpGCjT]T

where qGCj denotes the rotation quaternion from {G} to {C} and pGCj is the position of {C} in {G}.

According to Equation (1) and Equation (2), the full state vector with N camera states at time $t_{k}\in \left[\begin{array}{cc} t_{j} & t_{j+1} \end{array}\right)$ is given by,

(3) Xk=[XIkTqGCj−N+1TpGCj−N+1TqGCj−N+2TpGCj−N+1T⋯qGCjTpGCjT]T

Following Equation (3), the full error state ${\tilde{X}}_{k}\in {\mathbb{R}}^{21+6N}$ at time $t_{k}$ is defined as,

(4) X˜k=[X˜IkTθ˜GCj−N+1Tp˜GCj−N+1Tθ˜GCj−N+2Tp˜GCj−N+2T⋯θ˜GCjTp˜GCjT]T

where,

(5) X˜Ik=[θ˜GIkTb˜g,kTv˜GIkTb˜a,kTp˜GIkTθ˜ICTp˜ICT]T

For modeling the process model and state augmentation, we follow [22].

### 2.3. Measurement Model

#### 2.3.1. Vehicle Relative Rotation and Translation Estimation

**Figure 2:**
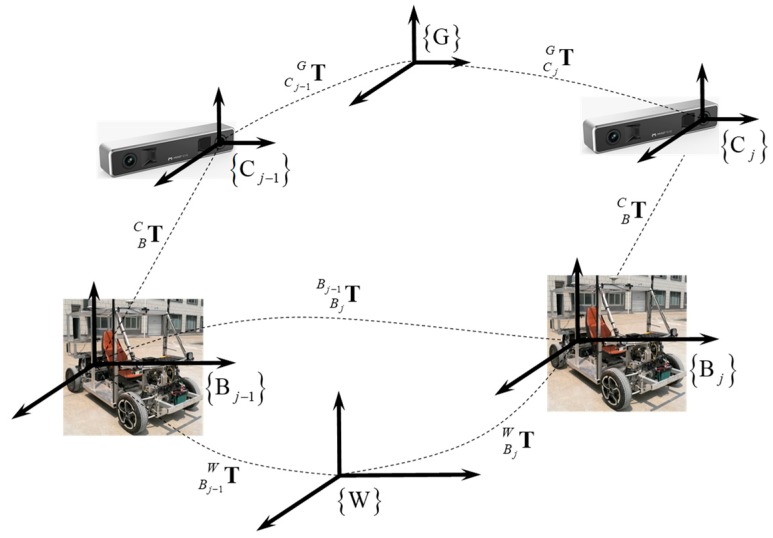
Geometric relation between {B} and {C} frames from time $t_{j-1}$ to time $t_{j}$. The symbol TBj−1W denotes the vehicle pose at time $t_{j-1}$ in {W}, TBjW denotes the vehicle pose at time $t_{j}$ in {W},TBjBj−1 represents the homogeneous coordinate transformation from time $t_{j}$ to time $t_{j-1}$, TBC represents the homogeneous coordinate transformation from {B} to {C}, TCj−1G denotes the camera pose at time $t_{j-1}$ in {G} and finally TCjG denotes the camera pose at time $t_{j}$ in {G}.

From the geometric constraints depicted in Figure 2, the estimation of vehicle relative rotation and translation can be given by the following equation:

(6) TBjBj−1=TBC−1TCj−1G−1TCjGTBC

Equation (6) can be represented as,

(7) C(qBjBj−1)=C(qBC)TC(qGCj−1)C(qGCj)TC(qBC)pBjBj−1=C(qBC)T[C(qGCj−1)[C(qGCj)TpBC+pCjG−pCj−1G]−pBC]

where,

(8) C(qBC)T=C(qIB)C(qIC)TpBC=pIC+pBI

where C(⋅) denotes the function converting the quaternion to the rotation matrix [41]. C(qIB) and pBI denote rotation extrinsic parameters and translation extrinsic parameters between {B} and {I}, respectively. C(qIC) and pIC denote rotation extrinsic parameters and translation extrinsic parameters between {C} and {I}, respectively. C(qBjBj−1) and pBjBj−1 are vehicle relative rotation and translation estimation from time $t_{j}$ to $t_{j-1}$.

#### 2.3.2. Ackermann Error State Measurements

After the state was augmented with the new camera state, the state is successively updated by Ackermann measurements and visual measurements. Here, the tightly-coupled Ackermann error state measurement model is present as the primary contribution of this paper.

Based on Equation (7), the measurement residual $r_{}^{B_{j}}$ can be defined as,

(9) rBj=(rθj−1,jBrvjB)=(zθj−1,jB⋅C(qBjBj−1)−1zvjB−pBjBj−1/Δtj−1,j)

where $r_{\theta_{j-1,j}}^{B}$ and $r_{v_{j}}^{B}$ are the Ackermann steering measurement residual from time $t_{j-1}$ to time $t_{j}$ and the velocity measurement residual at time $t_{j}$ respectively, and $z_{\theta_{j-1,j}}^{B}$ and $z_{v_{j}}^{B}$ are the Ackermann steering measurement and velocity measurement, respectively. The camera sampling period $\Delta t_{j-1,j}=t_{j}-t_{j-1}$. We assume that the velocity measurement $z_{v_{j}}^{B}$ is constant for low-speed motion from time $t_{j-1}$ to time $t_{j}$.

Considering the existence of nonholonomic constraint for a ground wheeled vehicle [43], the vehicle velocity in the plane perpendicular to the moving direction is considered as zero mean with Gaussian white noise. So, the velocity measurement $z_{v_{j}}^{B}$ can be represented as,

(10) $$z_{v_{j}}^{B}={\left[\begin{array}{ccc} v_{x,j}^{B} & 0 & 0 \end{array}\right]}^{T}$$

where $v_{x,j}^{B}$ is the vehicle longitudinal velocity at time $t_{j}$.

**Figure 3:**
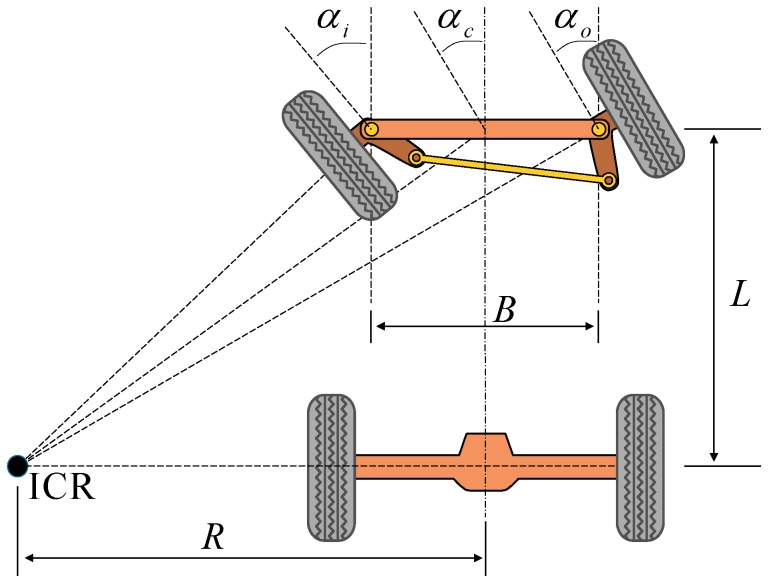
Ackermann steering geometry used in car-like wheeled mobile robots. B is the distance between the king pins, L is the wheel base (distance between the two axles), $\alpha_{i}$ is the steering angle of the inner wheel from straight ahead, $\alpha_{c}$ is the steering angle of the virtual wheel at the midpoint between the front wheels, $\alpha_{o}$ is the steering angle of the outer wheel from straight ahead and R is the steering radius (distance between the Instantaneous Center of Rotation (ICR) and the centerline of the vehicle). The sign of $\alpha_{i}$, $\alpha_{c}$, $\alpha_{o}$ and R follows the rule that a left turn assumes a positive value and a right turn a negative value in the coordinate system {B}.

The Ackermann steering measurement $z_{\theta_{j-1,j}}^{B}$ can be derived by the Ackermann steering geometry which is used in car-like wheeled mobile robots, and a closer look at the Ackermann steering geometry is illustrated in Figure 3. It is used to avoid wheel slippage by rotating the inner wheel with a larger angle than the outer wheel. As shown in Figure 3, the Instantaneous Center of Rotation (ICR) is located at the intersection of the extension cord of the automotive rear axle and the perpendicular bisectors of the front wheels [44].

It is assumed that vehicles follow the approximate planar motion, and there are no wheel slippage and sideslip angle for low-speed motion [45]. Therefore, the equation of Ackerman steering geometry then can be derived as follows,

(11) $$\begin{array}{c} \frac{L}{\tan\left|\alpha_{o}\right|}-\frac{L}{\tan\left|\alpha_{c}\right|}=\frac{B}{2}\\ R=\frac{L}{\tan\alpha_{c}} \end{array}$$

According to Equation (11), the kinematic relation between R and $\alpha_{o}$ is shown in:

(12) $$R=\{\begin{array}{l} L/\tan\alpha_{o}-B/2\;\mathrm{for}\;\alpha_{o}>0\\ L/\tan\alpha_{o}+B/2\;\mathrm{for}\;\alpha_{o}<0 \end{array}$$

(13) $$\alpha_{o}=\delta /\eta$$

where the steering wheel angle δ can be acquired from the vehicle CAN-bus. η is the angular transmission ratio obtained from the offline calibration in this paper, η=17.

By knowing the vehicle longitudinal velocity $v_{x}^{B}$, R and the vehicle CAN-bus sampling time $t_{\tau }$, the change of yaw angle $\phi_{j-1,j}^{B}$ from time $t_{j-1}$ to time $t_{j}$ is obtained [46]:

(14) $$\phi_{j-1,j}^{B}=\int_{\tau \in [j-1,j]}^{}v_{x,\tau }^{B}/R_{\tau }d\tau$$

Considering the nonholonomic constraint and the approximate planar motion assumption, the roll and pitch angle are considered as a zero mean with Gaussian white noise,

(15) $$\theta_{j-1,j}^{B}={\left[\begin{array}{ccc} 0 & 0 & \phi_{j-1,j}^{B} \end{array}\right]}^{T}$$

By using the Rodrigues rotation formula, the Ackermann steering measurement $z_{\theta_{j-1,j}}^{B}$ can be represented by the exponential map of $\theta_{j-1,j}^{B}$ [42]:

(16) $$z_{\theta_{j-1,j}}^{B}=\exp\left({\left(\theta_{j-1,j}^{B}\right)}^{\wedge }\right)$$

where the operator ${\left(\cdot \right)}^{\wedge }$ maps a vector to a skew symmetric matrix.

By linearizing Equation (9) at current error state ${\tilde{X}}_{k}$, $r_{}^{B_{j}}$ can be approximated as,

(17) $$r_{}^{B_{j}}=H_{}^{B_{j}}{\tilde{X}}_{k}+n_{}^{B_{j}}$$

where the Ackermann error state measurement Jacobian matrix $H_{}^{B_{j}}$ can be derived as,

(18) HBj=[03×15∂rθj−1,jB∂θ˜IC03×303×(6N−6)∂rθj−1,jB∂θ˜GCj03×303×15∂rvjB∂θ˜IC∂rvjB∂p˜IC03×(6N−6)∂rvjB∂θ˜GCj∂rvjB∂p˜GCj]

where

(19) ∂rθj−1,jB∂θ˜GCj=−C(qBC)TC(qGCj−1)C(qGCj)T∂rθj−1,jB∂θ˜IC=C(qBC)TC(qGCj−1)C(qGCj)T−C(qBC)T∂rvjB∂θ˜GCj=−C(qBC)TC(qGCj−1)C(qGCj)T⌊pBC×⌋/Δtj−1,j∂rvjB∂p˜GCj=C(qBC)TC(qGCj−1)/Δtj−1,j∂rvjB∂θ˜IC=C(qBC)T⌊C(qGCj−1)(pCjG−pCj−1G)×⌋/Δtj−1,j∂rvjB∂p˜IC=(C(qBC)TC(qGCj−1)C(qGCj)T−C(qBC)T)/Δtj−1,j

Meanwhile, $n_{}^{B_{j}}$ in Equation (17) denotes the noise of the measurement, and the measurement covariance matrix of $n_{}^{B_{j}}$ is given by,

(20) $$S_{}^{B_{j}}=\left[\begin{array}{cc} S_{\theta_{j-1,j}}^{B} & 0_{3\times 3}\\ 0_{3\times 3} & S_{v_{j}}^{B_{}} \end{array}\right]$$

where $S_{v_{j}}^{B}=\mathrm{diag}\left\{\begin{array}{ccc} \sigma_{v_{x}^{B}}^{2} & \sigma_{v_{y}^{B}}^{2} & \sigma_{v_{z}^{B}}^{2} \end{array}\right\}$, $S_{\theta_{j-1,j}}^{B}=\mathrm{diag}\left\{\begin{array}{ccc} \sigma_{\theta_{roll}^{B}}^{2} & \sigma_{\theta_{pitch}^{B}}^{2} & S_{\phi_{j-1,j}}^{B} \end{array}\right\}$, and $S_{\phi_{j-1,j}}^{B}$ can be given by the following recursive formula,

(21) $$S_{\phi_{j-1,\tau }}^{B}=S_{\phi_{j-1,\tau -1}}^{B}+V_{\tau -1}MV_{\tau -1}^{T}$$

where the input Jacobian matrix $V_{\tau -1}$ can be obtained according to Equation (14),

(22) $$\begin{array}{l} \phi_{j-1,\tau }^{B}=\phi_{j-1,\tau -1}^{B}+\Delta t_{\tau -1,\tau }v_{x,\tau -1}^{B}/R_{\tau -1}\\ V_{\tau -1}=\frac{\partial \phi_{j-1,\tau }^{B}}{\partial {\left[\begin{array}{cc} v_{x,\tau -1}^{B} & \delta_{\tau -1} \end{array}\right]}^{T}}=\left[\begin{array}{cc} V_{v_{x,\tau -1}^{B}}^{\phi } & V_{\delta_{\tau -1}}^{\phi } \end{array}\right] \end{array}$$

where $\Delta t_{\tau -1,\tau }=t_{\tau }-t_{\tau -1}$ is the vehicle CAN-bus sampling period, $V_{v_{x,\tau -1}^{B}}^{\phi }$ and $V_{\delta_{\tau -1}}^{\phi }$ can be derived as,

(23) $$\begin{array}{l} V_{v_{x,\tau -1}^{B}}^{\phi }=\frac{\Delta t_{\tau -1,\tau }}{R_{\tau -1}}\\ V_{\delta_{\tau -1}}^{\phi }=\frac{Lv_{x,\tau -1}^{B}\Delta t_{\tau -1,\tau }\left(\tan^{2}\left(\delta_{\tau -1}/\eta \right)+1\right)}{\eta {\left(R_{\tau -1}\tan\left(\delta_{\tau -1}/\eta \right)\right)}^{2}} \end{array}$$

and the input noise M is given by,

(24) $$M=\left[\begin{array}{cc} \sigma_{v_{x}^{B}}^{2} & 0\\ 0 & \sigma_{\delta }^{2} \end{array}\right]$$

where $\sigma_{v_{x}^{B}}$ and $\sigma_{\delta }$ are the standard deviations of vehicle longitudinal velocity and steering wheel angle, respectively. Meanwhile, $\sigma_{v_{y}^{B}}^{}$ and $\sigma_{v_{z}^{B}}^{}$ are the white-noise standard deviations of vehicle lateral velocity and vertical velocity, respectively. Moreover, $\sigma_{\theta_{roll}^{B}}$ and $\sigma_{\theta_{pitch}^{B}}$ are the white-noise standard deviations of vehicle roll angle and pitch angle, respectively. In this paper, $\sigma_{v_{x}^{B}}=0.3$, $\sigma_{v_{y}^{B}}^{}=0.3$, $\sigma_{v_{z}^{B}}^{}=5$, $\sigma_{\delta }=17$, $\sigma_{\theta_{roll}^{B}}=10\cdot \sqrt{S_{\phi_{j-1,j}}^{B}}$, $\sigma_{\theta_{pitch}^{B}}=10\cdot \sqrt{S_{\phi_{j-1,j}}^{B}}$.

Finally, the Kalman gain, updated state vector and covariance matrix at time $t_{j}$ can be obtained as follows,

(25) $$\begin{array}{l} K=P_{k|k}{\left(H^{B_{j}}\right)}^{T}{\left(H_{}^{B_{j}}P_{k|k}{\left(H^{B_{j}}\right)}^{T}+S_{}^{B_{j}}\right)}^{-1}\\ X_{k}^{+}=X_{k}+Kr_{}^{B_{j}}\\ P_{k|k}^{+}=(I_{\xi }-KH^{B_{j}})P_{k|k}{\left(I_{\xi }-KH^{B_{j}}\right)}^{T}+KS_{}^{B_{j}}K_{}^{T} \end{array}$$

where $I_{\xi }\in {\mathbb{R}}^{\left(21+6N\right)\times \left(21+6N\right)}$ represents the identity matrix.

#### 2.3.3. Visual Measurements

In the visual front-end, the FAST feature detector and KLT optical flow tracker are used. The configuration of the visual front-end and the visual feature measurement model in this paper are the same as S-MSCKF. Please refer to [22] for further details.

### 2.4. Extrinsic Parameters Calibration

Accurate extrinsic parameters can greatly affect the accuracy and reliability of overall multi-sensors’ fusion system performances. In this paper, the rotation extrinsic parameters C(qIC) and translation extrinsic parameters pIC between {C} and {I} are calibrated by using the Kalibr visual-inertial calibration toolbox [47]. The translation extrinsic parameters pBI between {B} and {I} are obtained by the vehicle design model, and we adopt the singular value decomposition (SVD) [48] rotation calibration and weighted average quaternion method [49] to calibrate rotation extrinsic parameters C(qIB) between {B} and {I}. A dual-antenna Spatial NAV982-RG inertial navigation system with real-time kinematic (GPS-RTK) is used to assist calibration. The GPS-RTK is fixed just above the automotive rear axle center and has the same axes as the {B}. Assuming that the installation error can be ignored, rotation extrinsic parameters C(qIB) can be obtained by aligning two rotation sequences from GPS-RTK and frame {I}. The following equation holds for any γ,

(26) qBγBγ−1⊗qIB=qIB⊗qIγIγ−1

where qBγBγ−1 and qIγIγ−1 are unit quaternions of GPS-RTK rotation measurements and gyroscope integral measurements of frame {I} from time $t_{\gamma }$ to time $t_{\gamma -1}$ respectively, and ⊗ is the quaternion multiplication operator.

Equation (26) can be represented as,

(27) [ΩL(qBγBγ−1)−ΩR(qIγIγ−1)]⋅qIB=Ωγγ−1⋅qIB=0

where

(28) ΩL(qBγBγ−1)=[qBγBγ−1w−qBγBγ−1xyzTqBγBγ−1xyzqBγBγ−1wI3+⌊qBγBγ−1xyz×⌋]ΩR(qIγIγ−1)=[qIγIγ−1w−qIγIγ−1xyzTqIγIγ−1xyzqIγIγ−1wI3−⌊qIγIγ−1xyz×⌋]

where ΩL(qBγBγ−1) and ΩR(qIγIγ−1) are matrix representations for left and right Hamilton [42] quaternion multiplication, and $q_{w}$ and $q_{xyz}$ are the real part and imaginary part of a quaternion. respectively.

By collecting a period of rotation sequences, the overconstrained linear system can be represented as,

(29) [Ω10Ω21⋮Ωγγ−1]qIB=Ωγ⋅qIB=0

where time $t_{\gamma }$ keeps going until the rotation calibration is completed.

Equation (29) can be solved by using SVD. Then, the right unit singular vector corresponding to the smallest singular value of $\Omega_{\gamma }$ is the solution. We set a threshold of iterations $N_{s}$, and the rotation calibration process terminates if $\gamma >N_{s}$.

After n rounds of rotation calibration with random sampling of rotation sequences, the weighted average quaternion [50] is utilized to obtain rotation extrinsic parameters qIB,

(30) qIB=argmaxq∈S3qTWq

where $S^{3}$ denotes the unit 3-sphere, and W is given by,

(31) W=∑i=1nsecσi⋅qIBi⋅qIBiT

where qIBi denotes rotation calibration solution in the i^th^ round, ${}_{}^{\sec}\sigma_{i}$ is the second smallest singular value of $\Omega_{\gamma }$ in the i^th^ round.

Equation (30) can be solved by using SVD, the rotation extrinsic parameters qIB are the eigenvector of W corresponding to the maximum eigenvalue.

## 3. Experiments and Results

The experimental vehicle setup and experimental scenarios are first introduced in this section. Secondly, the experimental results are exhibited for different vehicle driving cycles.

### 3.1. Experimental Vehicle Setup

To the best of our knowledge, it would be impractical to get public datasets collected by Ackermann steering vehicles in an outdoor environment, which contains both vehicle CAN-bus data, visual data, IMU data and ground truth. The public datasets used for VO and VIO are known as the EuRoC MAV Dataset [51], RGBD SLAM Dataset [52], KITTI Dataset [53] and PennCOSYVIO Dataset [54]. The EuRoC MAV Dataset is mainly suitable for MAVs applications; the RGBD SLAM dataset only contains RGBD camera data; the PennCOSYVIO Dataset is collected by handheld devices; and the KITTI Dataset is acquired by an Ackermann steering vehicle. However, the data does not contain time synchronous vehicle CAN-bus data. Therefore, the performance of the proposed method is evaluated by collecting real-world data.

**Figure 4:**
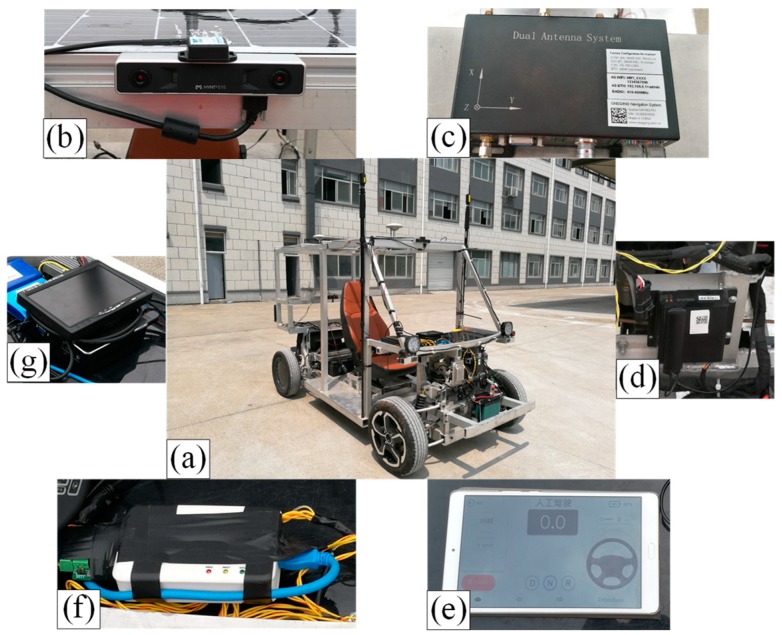
Experimental vehicle setup: (**a**) The Ackermann steering vehicle built by our research group; (**b**) MYNTEYE-S1030; (**c**) dual-antenna Spatial NAV982-RG; (**d**) telematic box ( (communicating with the smart phone application (APP) and Vehicle Control Unit (VCU)); (**e**) smart phone APP; (**f**) Controller Area Network (CAN) interface card; (**g**) mini-computer for the data acquisition.

The experimental vehicle is an Ackermann steering vehicle with dual in-wheel motors drive designed by our research group. As shown in Figure 4, the experimental vehicle can be remote controlled by using the smart phone application (APP) developed by our research group. Telematics BOX (T-BOX) is mainly used for communicating with the smart phone APP and Vehicle Control Unit (VCU) to realize the display and remote control of the experimental vehicle. The datasets were collected in the outdoor environment of the test field. We used the MYNTEYE-S1030 stereo camera (MYNTAI Inc., Beijing, China) with a focal length of 8 mm as the visual-inertial sensor. The camera with 720 pixels × 480 pixels resolution and 30 Hz frame rate is equipped with a time-synchronized IMU. The acquisition frequency of the IMU is 200 Hz. The vehicle longitudinal speed and steering wheel angle were acquired from the vehicle CAN-bus with sampling frequency 150 Hz by using the Controller Area Network (CAN) interface card.

A dual-antenna Spatial NAV982-RG inertial navigation system was used, which combines the FindCM positioning service from Qianxun Spatial Intelligence Inc. for centimeter-level positioning of the vehicle as the experimental ground truth.

The data acquisition was performed on a domestic mini-computer (Shenzhen Yingchi Technology Co., Ltd, Shenzhen, China) running on an Intel Core i3-5005U CPU with 2.00 GHz, 4 GB RAM (Intel, Santa Clara, CA, USA). The algorithm evaluation was performed on a Lenovo laptop (Lenovo, Beijing, China) running on an Intel Core i5-8300H CPU with 2.30 GHz, 8 GB RAM (Intel, Santa Clara, CA, USA). The data acquisition software and entire algorithm were implemented in C++ programming language (Free Software Foundation, Inc., Boston, Mass., USA) using ROS Kinetic (Open Source Robotics Foundation, Inc., Mountain View, CA, USA). We evaluated the trajectories using the methods provided by [55].

### 3.2. Experimental Results

**Figure 5:**
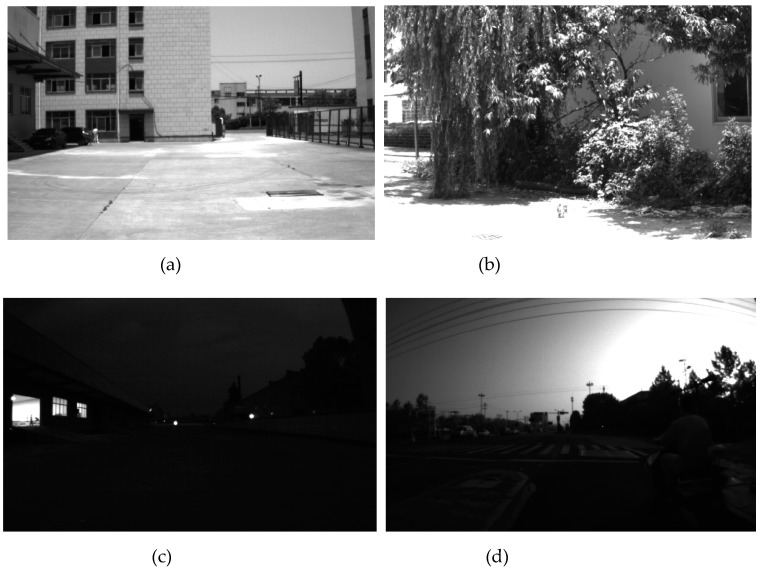
Acquired images in the experimental datasets. (**a**) AM_01; (**b**) AM_02; (**c**) AM_03; (**d**) AM_04.

Taking into account the different vehicle driving cycles and visually distinctive environmental features, in order to evaluate the algorithm more comprehensively, five different experimental datasets were acquired; i.e., the AM_01 dataset, AM_02 dataset, AM_03 dataset, AM_04 dataset and AM_05 dataset. AM_01 and AM_02 were respectively acquired under the straight line driving cycle and slalom line driving cycle by traveling around the building in a test field counterclockwise for one circle in the forenoon. In both of these datasets, there exist strong changes of illumination against the sunlight and motion blur at the sharp turns. AM_03 was acquired under the low-light environment at dusk which poses a great challenge for the VIO visual front-end. AM_04 with the dataset including road dynamic objects, such as other vehicles and pedestrians, was acquired under a long-distance driving cycle on the trafficway. AM_05 was acquired under a free driving cycle to verify the extrinsic parameters calibration method proposed in Section 2.4. The details of the experimental datasets are shown in Table 1, and Figure 5 shows several acquired images in the AM_01, AM_02, AM_03 and AM_04 datasets.

#### 3.2.1. Extrinsic Parameters Calibration Performance

The original S-MSCKF was utilized to evaluate the extrinsic parameters calibration performance on the AM_05 dataset. S-MSCKF with the calibration method proposed in Section 2.4 is named S-MSCKF(s), and S-MSCKF with its rotation extrinsic parameter manually measured is named S-MSCKF(m).

**Figure 6:**
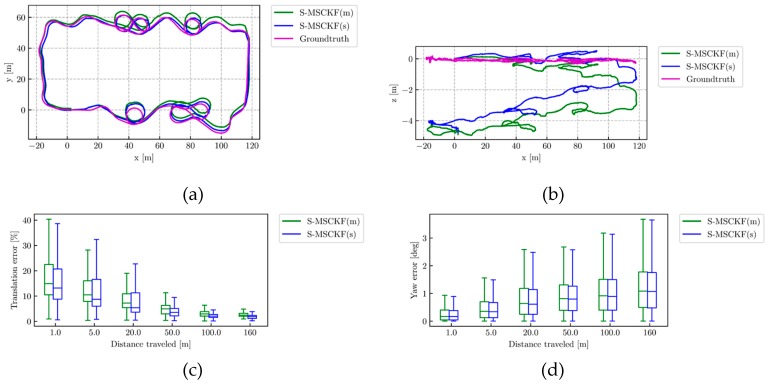
The performance from the Stereo Multi-State Constraint Kalman Filter (S-MSCKF)(s) (blue line), S-MSCKF(m) (green line) and ground truth (magenta line) on the AM_05 dataset. (**a**) Top-view of estimated trajectory; (**b**) Side-view of estimated trajectory; (**c**) The relative translation error; (**d**) The relative yaw error. Relative errors are measured over different sub-trajectories of length {1, 5, 20, 50, 100, 160} m.

Figure 6 shows that the performance from S-MSCKF(s) and S-MSCKF(m) on AM_05 dataset. For quantitative evaluation, we chose the absolute translation error (ATE) and the relative error (RE) as the evaluation metric. The open-source package [55] offers an off-the-shelf interface to perform a comprehensive evaluation of VIO. A yaw-only transformation with only the first state was used for trajectory alignment according to this package. Figure 6a,b show the top-view and side-view of the estimated trajectory, respectively. As presented in Figure 6c, the boxplots of the relative translation error are expressed in percentages by dividing the error with the sub-trajectory length. The boxplots of the relative yaw error are displayed in Figure 6d.

From Figure 6a, it can be seen that the estimated trajectory of S-MSCKF(s) aligns to the ground truth better than S-MSCKF(m). Obviously from Figure 6c, S-MSCKF(s) performs better than S-MSCKF(m) on relative translation error. Meanwhile, Figure 6b illustrates both methods exhibit large scale drift along the gravitational direction.

The absolute translation error on the AM_05 dataset is shown in Table 2, and the relative translation error and relative yaw error on the AM_05 dataset are shown in Table 3.

It can be seen from the quantitative results in Table 1 and Table 2 that the absolute translation error of S-MSCKF(s) is reduced by 27.55% of S-MSCKF(m). The relative translation error and relative yaw error of S-MSCKF(s) are less than S-MSCKF(m) over all sub-trajectory lengths. By comprehensive analysis of Table 1 and Table 2, S-MSCKF(s) performs better than S-MSCKF(m), indicating that the proposed extrinsic parameters’ calibration method can significantly improve the position accuracy.

#### 3.2.2. ACK-MSCKF Performance

The performance of ACK-MSCKF is compared with state-of-the-art open source stereo VIO, S-MSCKF and OKVIS on the AM_01, AM_02, AM_03 and AM_04 datasets acquired by our experimental vehicle. The extrinsic parameters obtained by our proposed calibration method are used for all of these algorithms.

**Figure 7:**
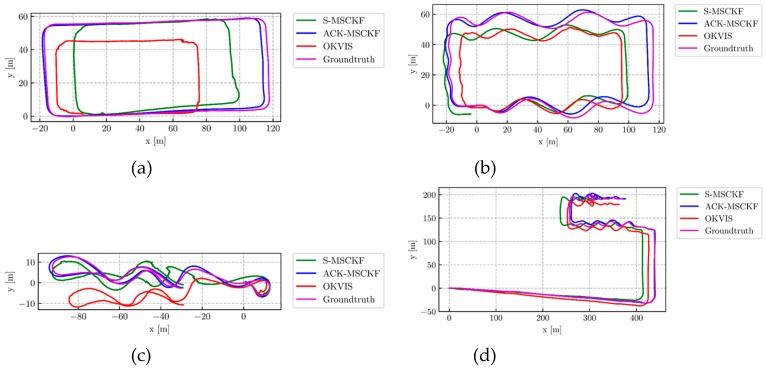
The top-views of estimated trajectory from ACK-MSCKF (blue line), S-MSCKF (green line), Open Keyframe-based Visual-Inertial SLAM (OKVIS) (red line) and ground truth (magenta line) of the dataset: (**a**) AM_01; (**b**) AM_02; (**c**) AM_03; (**d**) AM_04.

**Figure 8:**
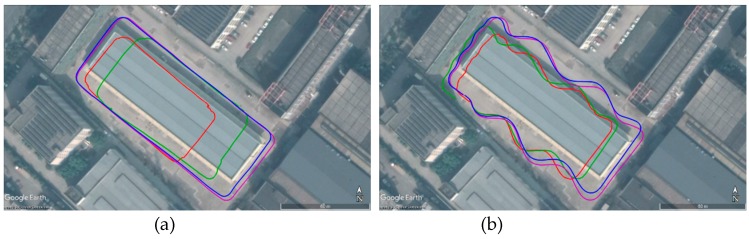

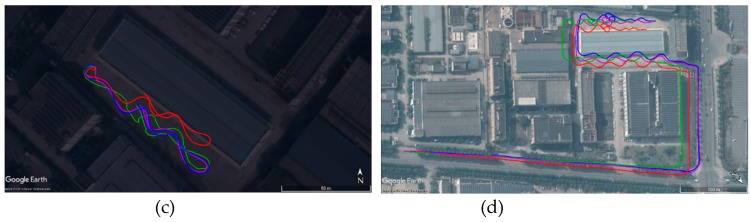
The estimated trajectories aligned to Google Earth from ACK-MSCKF (blue line), S-MSCKF (green line), OKVIS (red line) and ground truth (magenta line) of the dataset: (**a**) AM_01; (**b**) AM_02; (**c**) AM_03; (**d**) AM_04.

Figure 7 and Figure 8 show the trajectory estimation results in a top view and aligned to Google Earth, respectively.

From Figure 7 we can see that the estimated trajectories of our proposed ACK-MSCKF algorithm align to the ground truth better than others. Meanwhile, it can be seen that the position estimation results of S-MSCKF and OKVIS have larger scale drift, especially in the straight line driving cycle and slalom line driving cycle (Figure 7a,b). Figure 7c suggests that OKVIS has larger *y*-axis error in the low-light environment.

By fusing Ackermann error state measurements and S-MSCKF, it can be seen intuitively that the accuracy of the trajectory estimation is significantly improved.

Figure 8 shows that the trajectory of ground truth (magenta line) is highly identical with the outline of our test field in Google Earth. Therefore, the ground truth has high credibility to evaluate the performance of our algorithm.

**Figure 9:**
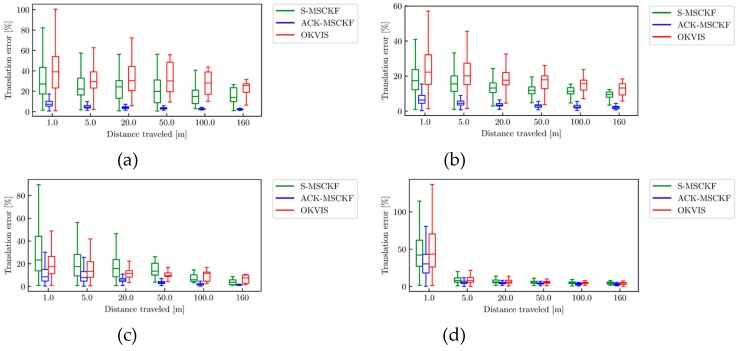
The relative translation error from ACK-MSCKF (blue line), S-MSCKF (green line) and OKVIS (red line) of the dataset: (**a**) AM_01; (**b**) AM_02; (**c**) AM_03; (**d**) AM_04.

**Figure 10:**
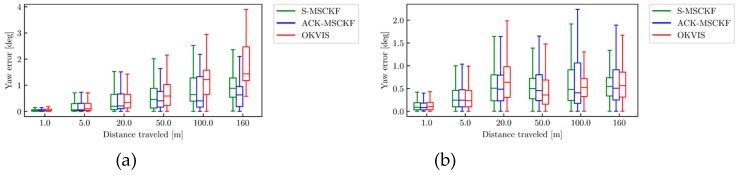

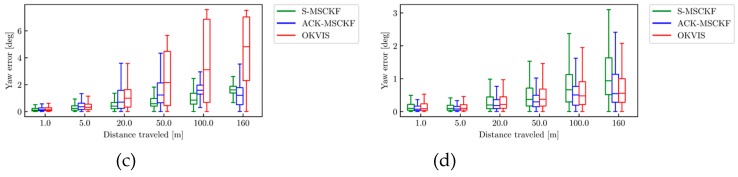
The relative yaw error from ACK-MSCKF (blue line), S-MSCKF (green line) and OKVIS (red line) of the dataset: (**a**) AM_01; (**b**) AM_02; (**c**) AM_03; (**d**) AM_04.

The boxplots of the relative translation error are shown in Figure 9. Figure 10 presents the boxplots of the relative yaw error.

Figure 9 demonstrates that the relative translation error of OKVIS on AM_01, AM_02 and AM_04 datasets is at the largest within the sub-trajectory length of 5 m, while S-MSCKF has the largest relative translation error within the sub-trajectory length of 50 m on the AM_03 dataset. Meanwhile, there exists larger scale drift, especially in the straight line driving cycle and slalom line driving cycle (Figure 9a,b).

It can be seen from Figure 10 that OKVIS has the largest relative yaw error within the sub-trajectory length of 20 m on all of datasets. The relative yaw error of OKVIS increases significantly along with the sub-trajectory length increases, especially in the straight line driving cycle and low-light environment (Figure 10a,c). S-MSCKF performs best on the relative yaw error within the sub-trajectory length of 100 m on the AM_03 dataset.

It can be concluded from Figure 9 and Figure 10 that the relative translation error of the ACK-MSCKF algorithm is significantly reduced, and the relative yaw error is close to S-MSCKF within the sub-trajectory length of 5 m.

The absolute translation error along the whole trajectory of each experimental dataset is shown in Table 4, and the overall relative translation error and relative yaw error on all of the real-world datasets are reflected in Table 5.

It can be seen from the quantitative results in Table 4 that the absolute translation error of ACK-MSCKF is the least on all of the datasets. There exists a larger absolute translation error, especially under the straight line driving cycle and outdoor long-distance driving cycle on the road. By analyzing the quantitative results in Table 5, it can be obtained that the relative translation error of S-MSCKF and OKVIS increases significantly along with the sub-trajectory length increases. At the same time, it illustrates that the relative yaw error of S-MSCKF is less than OKVIS over different sub-trajectory lengths.

By comprehensive analysis of Table 4 and Table 5, we can see that the relative translation errors of ACK-MSCKF in different sub-trajectory lengths are significantly reduced, and it is 4.18 m in the sub-trajectory length of 160 m, which is reduced respectively by 59.85% and 67.70% under the same condition of S-MSCKF and OKVIS. Meanwhile, the relative yaw error of ACK-MSCKF is close to S-MSCKF. The least relative yaw error from ACK-MSCKF is 0.80 deg in the sub-trajectory length of 160 m, which is reduced respectively by 23.08% and 39.85% under the same condition of S-MSCKF and OKVIS.

## 4. Discussion

From the experimental results, this can all be summarized as the following:

Accurate extrinsic parameters calibration greatly affects the accuracy and reliability of overall system performances. The extrinsic parameters calibration method proposed in this paper can significantly improve the position accuracy with the absolute translation error reduced by 27.55%.

S-MSCKF and OKVIS confront the problem of scale unobservability under special motions of autonomous vehicles. The reasons can be explained as follows. Visual features in outdoor scenes are mostly far away relative to the stereo baseline, making the stereo camera degenerated into a monocular one. Besides, the scale unobservability under special motions of ground vehicles attached to monocular VIO cannot be neglected.

ACK-MSCKF can achieve accurate and robust pose estimation under different vehicle driving cycles and environmental conditions, which mainly benefits from the improvement of observability under special motions of autonomous vehicles by fusing Ackermann error state measurements. The relative translation error of ACK-MSCKF is reduced respectively by 59.85% and 67.70% of S-MSCKF and OKVIS in the sub-trajectory length of 160 m. Moreover, the relative yaw error is reduced respectively by 23.08% and 39.85% under the same condition.

## 5. Conclusions

In conclusion, this paper proposes a tightly-coupled Ackermann visual-inertial odometry as ACK-MSCKF. The Ackermann steering geometry and Ackermann error state measurement model are illustrated in detail. Moreover, the rotation extrinsic parameters calibration method is presented. The performance is evaluated on real-world datasets acquired by an Ackermann steering vehicle. The experimental results demonstrate that the proposed ACK-MSCKF improves the pose estimation accuracy of S-MSCKF under special motions of autonomous vehicles, and can maintain the accuracy and robustness of pose estimation available under different vehicle driving cycles and environmental conditions. This work shows the guiding significance in the study of the high-precision pose estimation of Ackermann steering vehicles through an in-vehicle proprioceptive sensor and visual inertial sensor with a tightly-coupled filter-based mechanism.

However, the limitation of this work derives from the assumption that vehicles follow approximate planar motion without wheel slippage and sideslip angle for low-speed operation. In the future, we will further study the Ackermann error state measurement model-aided VIO under non-planar motion, and use incremental optimization-based algorithms [56,57,58,59] to further improve the pose estimation accuracy of autonomous vehicles.

## Figures and Tables

**Table 1:** List of our experimental datasets.

Dataset	Vehicle Driving Cycles	Environmental Condition	Duration (s)	Data Bulk			
				Vehicle CAN-Bus	Stereo Images	IMU	Ground Truth
AM_01	Straight	High-light	151	24,171	4532	30,164	30,244
AM_02	Slalom	Motion blur	170	27,135	5099	33,933	34,025
AM_03	Free	Low-light	131	17,387	3930	26,258	13,111
AM_04	Free	Dynamic objects	493	65,147	14,801	98,651	49,291
AM_05	Free	High-light	238	38,020	7136	47,439	47,583

**Table 2:** Absolute translation error on AM_05 dataset.

Methods	RMSE (m)
S-MSCKF(s)	3.05
S-MSCKF(m)	4.21

**Table 3:** The relative error on AM_05 dataset.

Sub-trajectory Length (m)	Relative Translation Error (m)		Relative Yaw Error (deg)	
	S-MSCKF(s)	S-MSCKF(m)	S-MSCKF(s)	S-MSCKF(m)
1	0.17	0.19	0.25	0.27
5	0.61	0.67	0.46	0.48
20	1.56	1.76	0.76	0.79
50	1.94	2.39	0.91	0.93
100	2.20	2.96	1.00	1.02
160	2.97	4.14	1.18	1.20

**Table 4:** Absolute translation error along the whole trajectory of each experimental dataset.

Methods	AM_01 (m)	AM_02 (m)	AM_03 (m)	AM_04 (m)
ACK-MSCKF	2.25	3.43	1.57	5.13
S-MSCKF	15.24	12.26	5.23	21.66
OKVIS	24.62	13.01	9.68	13.63

**Table 5:** The overall relative error on all of the real-world datasets.

Sub-trajectory Length (m)	Relative Translation Error (m)			Relative Yaw Error (deg)		
	ACK-MSCKF	S-MSCKF	OKVIS	ACK-MSCKF	S-MSCKF	OKVIS
1	0.25	0.41	0.46	0.14	0.15	0.36
5	0.34	0.75	0.80	0.21	0.20	0.41
20	0.97	2.48	2.65	0.43	0.39	0.68
50	1.85	4.99	5.88	0.59	0.55	0.87
100	2.99	7.70	9.99	0.74	0.78	1.11
160	4.18	10.41	12.94	0.80	1.04	1.33
